# Dynamic time window mechanism for time synchronous VEP-based BCIs—Performance evaluation with a dictionary-supported BCI speller employing SSVEP and c-VEP

**DOI:** 10.1371/journal.pone.0218177

**Published:** 2019-06-13

**Authors:** Felix Gembler, Piotr Stawicki, Abdul Saboor, Ivan Volosyak

**Affiliations:** Faculty of Technology and Bionics, Rhine-Waal University of Applied Sciences, 47533 Kleve, Germany; Columbia University, UNITED STATES

## Abstract

Brain-Computer Interfaces (BCIs) based on visual evoked potentials (VEPs) allow high communication speeds and accuracies. The fastest speeds can be achieved if targets are identified in a synchronous way (i.e., after a pre-set time period the system will produce a command output). The duration a target needs to be fixated on until the system classifies an output command affects the overall system performance. Hence, extracting a data window dedicated for the classification is of critical importance for VEP-based BCIs. Secondly, unintentional fixation on a target could easily lead to its selection. For the practical usability of BCI applications it is desirable to distinguish between intentional and unintentional fixations. This can be achieved by using threshold-based target identification methods. The study explores personalized dynamic classification time windows for threshold-based time synchronous VEP BCIs. The proposed techniques were tested employing the SSVEP and the c-VEP paradigm. Spelling performance was evaluated using an 8-target dictionary-supported BCI utilizing an n-gram word prediction model. The performance of twelve healthy participants was assessed with the information transfer rate (ITR) and accuracy. All participants completed sentence spelling tasks, reaching average accuracies of 94% and 96.3% for the c-VEP and the SSVEP paradigm, respectively. Average ITRs around 57 bpm were achieved for both paradigms.

## Introduction

Brain-Computer Interfaces (BCIs) detect, analyze, and decode brain activities to provide communication with the external environment, without involving any muscle activities [1]. The brain activities are usually recorded non-invasively by an electroencephalogram (EEG). BCIs may be used as a communication tool for severely impaired people [2, 3].

In comparison to other BCI paradigms, BCIs based on visual evoked potentials (VEPs) yield the fastest spelling performance [4–7]. VEPs are brain responses to a visual stimulus and they are usually categorized according to the type of the modulation stimulus. The most commonly used VEPs in BCI research are the frequency modulated steady-state visual evoked potentials (SSVEPs or f-VEPs) [5, 8, 9] and the code-modulated VEPs (c-VEPs) [7, 10, 11]. Recently, an information transfer rate (ITR) of 325.33 bpm has been achieved with a 40-target BCI using the SSVEP paradigm [5]. In the SSVEP paradigm, each target to be selected flashes at a specific frequency. When such a stimulus is fixated, continuous brain responses are elicited at the occipital and parietal cortical areas of the brain; the fundamental frequency of the stimulus as well as its harmonics can be detected.

In contrast, for c-VEP BCIs, all targets are modulated with different time lags of the same code sequence [12]. Usually pseudorandom m-sequences which have good autocorrelation properties are used [12].

During the last decades, the spelling interfaces are among the most widely utilized applications in BCI research [13]. To demonstrate usability and feasibility of such applications, various performance metrics such as the ITR can be applied [14]. Evaluation of BCI performance of such applications typically requires participants to type predefined words or phrases.

While SSVEP BCIs can be realized without training sessions, highest spelling speeds are achieved if pre-recorded user EEG data are used in a specific way to classify the attended target [15]. While this approach yields high ITRs, usually static classification time windows, which are dependent on the recording session, are used. For c-VEP-based BCIs, static time windows are the standard because of the required synchronization between EEG data collection and stimulus representation, and the fixed length of the code sequence; sliding EEG data windows are technically much harder to realize.

If the synchronous approach is utilized in VEP-BCIs (employing either SSVEP or c-VEP flashing patterns), the pre-recorded template with the maximum correlation to the recorded data determines the output command. Typically, applications introduce a flickering pause after a command is classified (flickering and, if applicable, data collection pause), which allows the user to shift the gaze to the next target. In the literature, this stimulation pause is often referred to as gaze shifting period [16], gaze shifting time or cue duration [17], break between trials [18], or rest period [19]. After this pre-set gaze shifting period, the stimuli continue flickering for exactly one stimulation cycle again, i.e. the user cannot influence the duration of the flickering. The BCI outputs are determined at equidistant time points. While this approach leads to high target selection speeds if short classification windows are used, it might be impractical in longterm daily use as it can easily lead to unintentional target activation.

Besides high responsiveness, a natural user-BCI interaction requires the system to distinguish between intentional and unintentional gaze fixation. Especially, during long term use, the user might need longer time to locate a desired target on the screen; for example, in spelling applications, the time the user needs to shift his/her gaze to a desired letter depends on many factors, such as familiarity and complexity of the letter arrangement. Complex user interface extensions like dictionary-based word suggestion mechanisms might update the selection options after every command [20]. Hence, a more flexible classification approach is needed. Therefore, we utilize flexible threshold-based classification time windows in this study.

For the SSVEP paradigm, such flexible classification times have been realized using classification thresholds [18, 21]. For instance, in the SSVEP-based Bremen BCI speller, the target activation corresponding to a specific frequency is only performed, if a pre-defined threshold is surpassed; otherwise, the classified command is rejected [21].

Classification thresholds make the system more robust, but can slow down the output information transfer rate, as it takes extra time until a threshold is surpassed. In spelling applications, this performance drop can be compensated using word prediction methods, which have been widely used in BCI research [20, 22]. Prediction methods based on *n*-gram models have only recently been realized in BCIs [23]. The *n*-gram model is used for the prediction of the next items in a sequence. Item probabilities are extracted from a text database. While it is typically used with individual characters as items in BCI, here, the *n*-gram model was utilized at the word level, i.e., the words with highest probability were suggested based on the previously entered text.

## Materials and methods

### Participants

Twelve healthy participants were recruited for this experiment, eight female and four male (average age 23.75 years, SD 2.35, range 21 to 30 years). All subjects had normal or corrected to normal vision. The research was approved by the ethical committee of the medical faculty of University Duisburg-Essen. Prior to the experiment, the participants were informed about the purpose, risks, and design of the study. The subjects who agreed to participate in the study signed an informed consent in accordance with the Helsinki declaration. Information needed for the analysis of the experiments was stored anonymously during the experiments. The participants had the opportunity to opt-out of the study at any time. All subjects received a financial reward for their participation.

### Hardware

The used computer (MSI GT 73VR with nVidia GTX1070 graphic card) operated on Microsoft Windows 10 Education running on an Intel processor (Intel Core i7, 2.70 GHz). A liquid crystal display screen (Asus ROG Swift PG258Q, 1920 × 1080 pixel, 240 Hz maximal refresh rate) was used.

An EEG amplifier (g.USBamp, Guger Technologies, Graz, Austria) was used, utilizing all its 16 signal channels. The 16 signal electrodes were placed according to the international 10/5 system of electrode placement (see, e.g., [24] for more details): P_Z_, P_3_, P_4_, P_5_, P_6_, PO_3_, PO_4_, PO_7_, PO_8_, POO_1_, POO_2_, O_1_, O_2_, O_Z_, O_9_, and O_10_. The reference electrode was placed at C_Z_ and the ground electrode at AF_Z_. Standard abrasive electrolytic electrode gel was applied between the electrodes and the scalp to bring impedances below 5 kΩ during the preparation phase. An analogue band pass filter (between 2 and 60 Hz) and a notch filter (around 50 Hz) were applied in the amplifier.

### Stimulus presentation

In this experiment, the number of stimulus classes, *K*, was set to 8. Therefore, a distinct flashing pattern was assigned to each target. Two stimulus types, SSVEP- and c-VEP stimuli, were tested consecutively.

#### c-VEP stimulus presentation

The target stimuli consisted of eight boxes (230 × 230 pixel) arranged as 2 × 4 stimulus matrix (see section Software for more details).

The c-VEP paradigm is often realized by employing the so-called m-sequences, non-periodic binary codes with good autocorrelation properties [12]. According to Wei et al. [10], a modulation sequence with a length of 63 bit and a lag of 4 bits between adjacent stimuli yields good performance.

Hence, for the flashing pattern 63 bit m-sequences *c*_*i*_, *i* = 1, …, *K* were assigned to the stimulus matrix employing a circular shift of 4 bits (*c*_1_ had no shift, *c*_2_ was shifted by 4 bits to the left, *c*_3_ was shifted by 8 bits, etc.). The codes were assigned row-wise to the matrix (i.e. starting from the upper left target with *c*_1_, further targets were assigned in row major order). The stimuli corresponding to the codes alternated between the states ‘black’ (the background color, represented by ‘0’) and ‘white’ (represented by ‘1’). Here, *c*_1_ was defined as

$$\begin{array}{c} c_{1}=101011001101110110100100111000101111001010001100001000001111110. \end{array}$$

The duration of a stimulus cycle in seconds can be calculated by dividing the code length by the monitor refresh rate *r* in Hz; in this experiment, 63/60 = 1.05*s*.

#### SSVEP stimulus presentation

For the SSVEP flashing pattern, a specific frequency *f* and phase *Φ* were assigned to each target [25]. The flickering was realized by sinusoidally modulating their transparencies in accordance with the frequency/phase combination, as described e.g. in [26, 27].

For this, the stimulus sequence for the *i*-th target is calculated as follows:

$$\begin{array}{c} c_{i}\left(t\right)=\frac{1}{2}(1+\sin(2\pi f_{i}\frac{t}{r}+\Phi_{i})),\;t=0,1,\ldots , \end{array}$$ (1)

yielding values in the range from 0 to 1. Alpha compositing, i.e., the combination of an image with the background, was utilized to modulate the transparency of the stimulus according to this sequence. The alpha channel of the RGBA color space indicates how opaque a pixel is. An alpha value of zero, *α* = 0, corresponds to full transparency and *α* = 255 corresponds to no transparency. A black background was used for the stimuli. The color value of a stimulus was set to RGBA = (255, 255, 255, *α*), where alpha was set to *c*_*i*_(*t*) * 255. As a black background was used, this resulted in the target color ‘black’ if *c*_*i*_(*t*) = 0 and ‘white’ if *c*_*i*_(*t*) = 1.

Similar to [26], frequencies *f*_*i*_ = *f*_0_ + Δ*f* and phases Φ_*i*_ = Φ_0_ + ΔΦ, *i* = 1, …, *K*, with *f*_0_ = 8 Hz, Δ*f* = 1 Hz, Φ_0_ = 0 and ΔΦ = 0.35*π*, where assigned column-wise to the stimulus matrix.

This frequency range was chosen, as it avoids mutual influences between fundamental and harmonic frequencies. Further, due to the 1-Hz difference between stimuli, the stimulus cycle is of length *r*, in other words, the repetition period is 1 s.

### Experimental protocol

Participants sat on a chair facing the LCD screen (at a distance of approximately 60 cm). After they were prepared for the EEG recording, they went through two sessions (c-VEP and SSVEP). Each session consisted of a training phase (for template recording and automated parameter setup), an on-line copy spelling phase, and a brief questionnaire. The experiment took approximately one hour for each participant. The order of the starting paradigm was altered for every other participant. Hence half of the participants started the experiment with the SSVEP paradigm, the other half with the c-VEP paradigm.

#### Training phase

In the training phase, each of the eight stimuli was fixated several times. For each trial the code pattern repeated for three cycles, i.e., the stimuli flickered for 3 * 1 = 3*s* for the SSVEP paradigm and for 3 * 1.05 = 3.15*s* for the c-VEP paradigm. A green frame around the box indicated which box the user had to fixate. Initially, subjects pressed the space bar to start the stimulation.

The recording was grouped in six training blocks, *n*_*b*_ = 6. In each block every stimulus was attended once, resulting in 6 * 8 = 48 trials in total. After each trial, the next box was highlighted, the flickering paused for one second, and the participant shifted his/her gaze to the next target.

In order to avoid visual fatigue, subjects were allowed to take breaks after each block of eight trials (the recording automatically paused). To start another recording block, the subjects needed to click the space bar.

#### Copy spelling phase

Prior to the copy spelling task, a brief familiarization run was performed were participants spelled the word KLEVE, and a word of free choice (e.g. the own first name). During this familiarization run, in some cases, the automatically determined classification thresholds were lowered manually to increase the responsiveness of the application. In the copy spelling phase, participants were asked to spell the words BCI and BRAIN as well as a longer English sentence. For each participant and paradigm, different sentences were used. Occurring errors were corrected using the UNDO function of the interface.

#### Questionnaires

Prior to the training phase, participants filled in a brief questionnaire, answering questions regarding gender and age. Additionally, after each session, participants gave their subjective impressions of the BCI answering questions regarding fatigue and annoyance. The questions as well as the collected answers of this questionnaires are provided in the results section.

### Software

#### CCA-based spatial filters

Canonical-correlation analysis (CCA) is a statistical method which investigates the relationship between two sets of variables [28]. Given two multidimensional variables $\text{X}\in R^{p\times s}$ and $Y\in R^{q\times s}$, CCA finds weight vectors $w_{X}\in R^{p}$ and $w_{Y}\in R^{q}$ that maximize the correlation *ρ* between the linear combinations **x** = **X**^*T*^**w**_**X**_ and **y** = **Y**^*T*^**w**_**Y**_. The weight vectors **w**_**X**_ and **w**_**Y**_ are determined by solving

$$\begin{array}{c} \max_{w_{X},w_{Y}}\rho \left(x,y\right)=\frac{E[w_{X}^{T}XY^{T}w_{Y}]}{\sqrt{E\left[w_{X}^{T}XX^{T}w_{Y}\right]E\left[w_{Y}^{T}YY^{T}w_{Y}\right]}} \end{array}$$ (2)

The value *ρ* is the first and maximal canonical correlation and **x** and **y** represent the first pair of canonical variables. Successively, additional pairs **x**, **y** can be constructed by maximizing (2) with the constraint that they are orthogonal to the already found pairs. The number of canonical variable pairs is constrained by the dimensionality of the variables **X** and **Y**. In BCI research, CCA is used to find a linear transformation that maximizes the correlation between the recorded signal and the averaged template signals. Typically, only the first canonical correlation and corresponding weight is used for classification and construction of filters [4, 5, 29]. Nonetheless, some recent studies yielded better performance when the additional canonical variable pairs with their associated correlations and weights where also utilized [30].

Here, to improve signal-to noise ratio, CCA was used to create class-specific spatial filters (see, e.g., [5, 7]).

In this regard, filters were constructed from the training data as follows: The trials recorded during the training phase were stored in a *m* × *n*_*t*_ matrix, where *m* denotes the number of electrode channels (here *m* = 16) and *n*_*t*_ denotes the number of samples. All *n*_*b*_ trials $T_{i}\in R^{m\times n_{t}}$ corresponding to a specific class are averaged by calculating the arithmetic mean, yielding the template $X\in R^{m\times n_{t}}$,

$$\begin{array}{c} X=\frac{1}{n_{b}}\sum_{i=1}^{n_{b}}T_{i}. \end{array}$$

For the filter, two additional matrices need to be constructed. All trials **T**_*i*_ are concatenated horizontally yielding a matrix $\hat{T}\in R^{m\;\times \;(n_{b}n_{t})}$,

$$\begin{array}{c} \hat{T}=\left[T_{1}T_{2}\ldots T_{n_{b}}\right]. \end{array}$$

A second matrix $\hat{X}$ with the same dimensions is constructed by replicating **X**,

$$\begin{array}{c} \hat{X}=\left[\underset{n_{b}}{\underset{︸}{XX\ldots X}}\right]. \end{array}$$

By plugging $\hat{X}$ and $\hat{T}$ into Eq (2), the weight vector $w=w_{\hat{X}}$ is obtained.

This procedure is applied for all classes, yielding a set of training templates **X**_*i*_ and weights **w**_*i*_, *i* = 1, …, *K*.

#### Time window mechanism

The output command corresponding to a classification was only performed if certain thresholds were surpassed. In this regard, sliding classification time windows of dynamic length were utilized [8], i.e., in the case where no classification could be made, a new classification was performed, after receiving the new EEG data.

The multichannel EEG signals that were about to be classified were stored in a matrix $Y\in R^{m\times n_{y}}$, where *n*_*y*_ represents the length of the classification time window in samples. In praxis, *n*_*y*_ needs to be selected carefully. Too small time windows can lead to errors [31]. On the other hand, if *n*_*y*_ is too large, data unrelated to the desired target (e.g. due to gaze movements at the beginning of the time window) remains to be considered for classification, which can slow down performance. Therefore, *n*_*y*_ needs to be restricted,

$$\begin{array}{c} {n_{y}}_{\min}\leq n_{y}\leq {n_{y}}_{\max}. \end{array}$$ (3)

The selection of the minimum time window, the lower bound in (3), is critical. Here, it was determined individually based on the training data as elaborated later. Recall that the stimulus cycle was 1 s and 1.05 s for the SSVEP and c-VEP approach respectively. The number of samples collected in this period, *n*_*c*_, was therefore *n*_*c*_ = 600 for SSVEP and and *n*_*c*_ = 630 for c-VEP. The upper bound, *n*_*y*__max_, was selected as a multiple of *n*_*c*_.

In the on-line BCI, the time window extended incrementally. The amplifier transfers EEG data in blocks $A_{i}\in R^{m\times n_{a}}$, where *n*_*a*_ denotes the number of samples per block. For the implementation of the sliding window mechanism, *n*_*a*_ was selected as divider of the cycle length, i.e., *n*_*a*_|*n*_*c*_.

A further restriction to *n*_*a*_ was given by the amplifier manufacturer. For the gUSBamp, the buffer needed to contain at least 20-30 ms of data. Here, *n*_*a*_ was set to 30 samples (50 ms recordings with the sampling rate of 600 Hz).

The amplifier blocks where accumulated in a buffer $\hat{A}\in R^{m\times n_{\hat{a}}}$,

$$\begin{array}{c} \hat{A}=\left[A_{1},A_{2},\ldots \right]. \end{array}$$

If the number of samples of the buffer, $n_{\hat{a}}$, was to small, i.e., $n_{\hat{a}}<{n_{y}}_{\min}$, no classification was performed.

If ${n_{y}}_{\min}\leq n_{\hat{a}}\leq {n_{y}}_{\max}$, the classification time window gradually increased (with step width *n*_*a*_). The classification was performed using the data matrix $Y=\hat{A}$, i.e., all data from the buffer were considered for classification. If the classifier did not meet a certain threshold criterion, as described later, further EEG data was collected.

Lastly, if $n_{\hat{a}}>{n_{y}}_{\max}$, only the last *n*_*y*__max_ samples were used for classification. Therefore, we set **Y** as the sub-matrix of $\hat{A}$ formed from rows 1, …, *m* and columns $n_{c}k+1,\ldots ,n_{\hat{a}}$,

$$\begin{array}{c} Y=\hat{A}\left[1,\ldots ,m\;;\;n_{c}k+1,\ldots ,n_{\hat{a}}\right], \end{array}$$

where *k* is the smallest integer such that *n*_*y*_ follows (3). Since *n*_*a*_|*n*_*c*_, data collection and stimulus presentation remain synchronized.

#### Classification

The test matrix $Y\in R^{m\times n_{y}}$ is compared against individual reference templates $R_{i}\in R^{m\times n_{y}}$, *i* = 1, …, *K* each of which was set as the sub-matrix of the corresponding training template **X**_*i*_, formed from rows 1, …, *m* and columns 1, …, *n*_*y*_,

$$\begin{array}{c} R_{i}=X_{i}\left[1,\ldots ,m\;;\;1,\ldots ,n_{y}\right]. \end{array}$$ (4)

As the spatial filters for each target are similar to each other, we adopted the ensemble-based target identification which was proposed originally for the SSVEP paradigm in [5]. Ensemble correlations, λ_*k*_, were determined by stacking all target-specific spatial filtered data and template vectors as follows:

$$\begin{array}{c} \lambda_{k}=\rho \;([\begin{array}{c} Y^{T}w_{1}\\ \vdots \\ Y^{T}w_{K} \end{array}],[\begin{array}{c} {R_{k}}^{T}w_{1}\\ \vdots \\ {R_{k}}^{T}w_{K} \end{array}],\;k=1,\ldots ,K. \end{array}$$ (5)

Additionally, the difference between target- and non-target correlations can be enhanced further by applying a filter bank method, which decomposes VEP-data in sub-band components as described in [17]. The lower and upper cut-off frequencies for the *m*-th sub-band were selected as *m* * 8 and 60 Hz. To this end, an 8th order Butterworth filter was utilized. Forward and reverse filtering were applied to cancel the phase response [32].

The ensemble approach, Eq (5), was then applied to each sub-band component individually, yielding a set of correlations $\lambda_{k}^{\left(1\right)},\lambda_{k}^{\left(2\right)},\ldots ,\lambda_{k}^{\left(M\right)}$, *k* = 1, …, *K*, where *M* denotes the number of considered sub-bands. Then, the output command candidate was determined using weighted linear combinations of the correlations,

$$\begin{array}{c} C=\underset{k=1,\ldots ,K}{arg max}{\tilde{\lambda }}_{k},\;\text{where}\;{\tilde{\lambda }}_{k}=\sum_{m=1}^{M}a_{m}\lambda_{k}^{\left(m\right)}. \end{array}$$ (6)

Mirroring the decrease in amplitude in the higher bands, the weights *a*_*m*_ in (6) where set as

$$\begin{array}{c} a_{m}=\frac{a_{m}^{\prime }}{\sum_{k=1}^{M}a^{\prime }},\;\text{with}\;a_{m}^{\prime }=m^{-1.25}+0.25, \end{array}$$ (7)

yielding decreasing weights for the higher bands. The optimal choice of these weights needs to be investigated further (see, e.g. [17]).

The number of sub-bands, *M*, was set to 1 and 5, for c-VEP and SSVEP, respectively. In the on-line copy spelling phase, the action associated with a classified label *C* was only performed if a certain threshold criterion was met, which is described in the following. The decision certainty, Δ_*C*_, was calculated as the distance between the highest and second highest correlation. The output was performed if Δ_*C*_ exceeded a certain threshold value, *β*; otherwise the, classifier output was rejected. In other words, the output command was only performed if *n*_*y*_ ≥ *n*_*y*__min_ and Δ_*C*_ ≥ *β*.

After a produced output command, the data buffers *B* and *Y* were cleared and a 1 second gaze shifting period followed. In this gaze shifting period, the amplifier data blocks, *A*_*i*_, were ignored and the stimuli did not flicker, allowing the user to shift his/her gaze to the next target. Please note that the BCI did not require a full cycle of the stimulation pattern for classification. If a command was classified before the stimulus pattern completed a full cycle (1.05 s for c-VEP and and 1 s SSVEP), the flickering stopped.

#### Automatic parameter calibration

In order to realize individually optimized system parameters, the values for the classification threshold *β* and for the minimum time window *n*_*y*__min_ were determined automatically for each participant on the basis of the training data via a leave-one-out cross-validation.

In this regard, we considered the ITR in bpm (see [1], an on-line calculation tool can be found at https://bci-lab.hochschule-rhein-waal.de/en/itr.html),

$$\begin{array}{c} ITR=\frac{\log_{2}K+p\log_{2}p+\left(1-p\right)\log_{2}(\frac{1-p}{K-1})}{t/60}, \end{array}$$ (8)

where the target identification accuracy, *p*, was calculated based on the number of correctly classified commands divided by the total number of commands, and *t* represented the average time needed to make a selection (in s).

**Fig 1:**
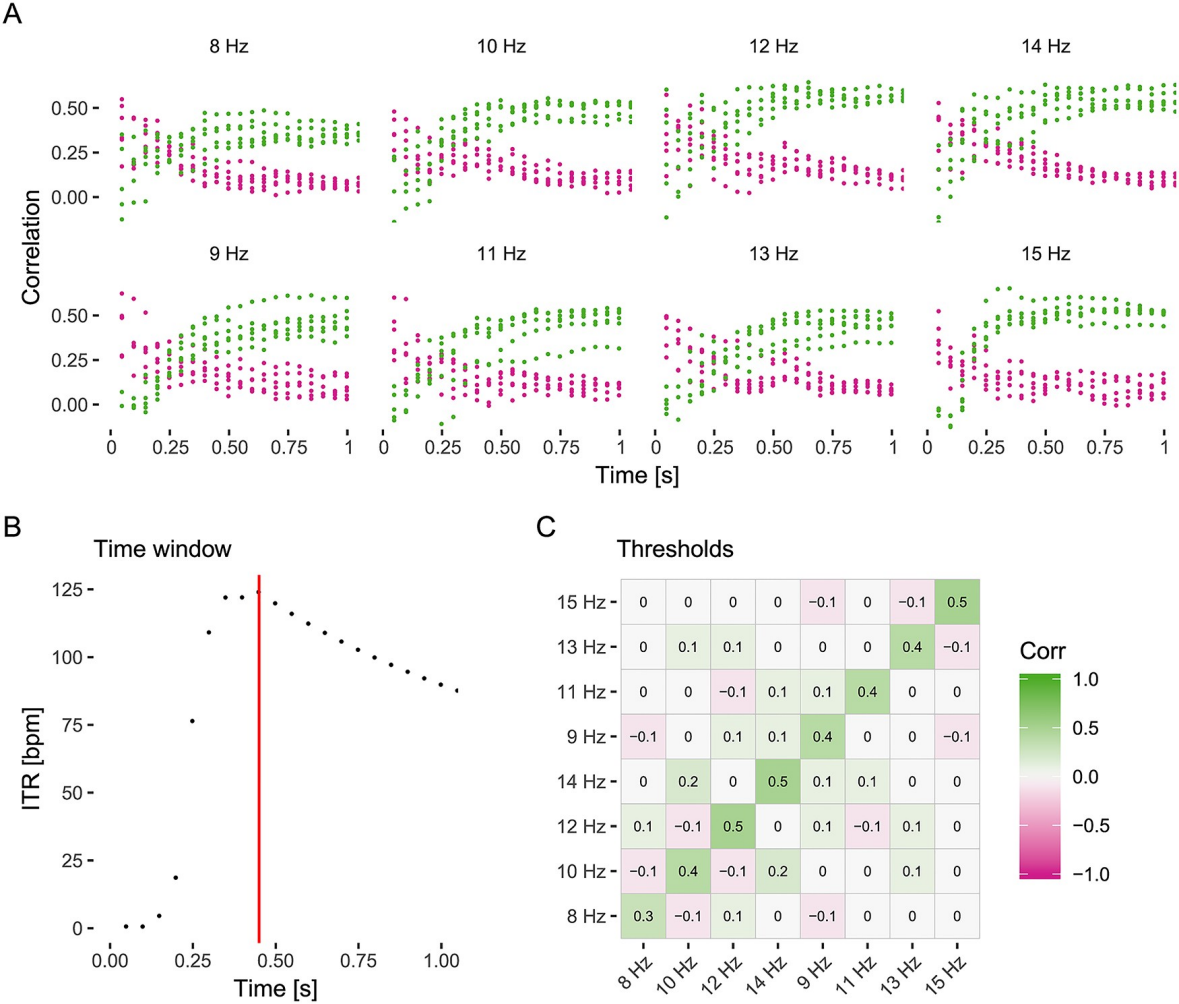
Example of the proposed automatic selection of the classification parameters. Displayed are the results from the leave-one-out off-line analysis of the SSVEP training data for one participant. In the training session, each of the 8 targets was attended 6 times for 3 seconds. (A) Displayed are the correlation values of the target stimulus (green) and the maximum correlation of the non-target stimuli (red). For each stimulus class the correlations calculated in the first second are displayed. (B) ITR averaged over the 6 training blocks. Here, the time window yielding the highest ITR was 0.45 seconds. (C) Correlogram of the training data. Depicted are the correlations for each target using the determined time window, averaged over the trials. This minimal difference between target and non-target stimuli was used as the threshold value. Here, the distance was minimal for the 10 and 14 Hz pair, yielding a difference of 0.2.

Utilizing leave-one-out cross-validation on the training data, an average ITR was calculated for classification windows of *n*_*y*_ = 30, 60, …, 3*n*_*c*_ samples. The value of *n*_*y*_ that maximized the ITR (8) was selected as minimum time window *n*_*y*__min_. The classification threshold *β* was selected as the minimal decision certainty, Δ_*C*_, at that time window. An example of the parameter setup procedure is depicted in Fig 1.

As stated in the experimental protocol, the suggested thresholds were sometimes lowered manually in the on-line spelling tasks. An explanation for the sometimes lower certainty in on-line tasks is that in contrast to the cue-guided training, it was not guaranteed that participants were fixating the target when the flickering started.

#### Dictionary-driven spelling application

The spelling application utilized *n*-gram prediction, which is used in computational linguistics. An *n*-gram describes a sequence of *n* items from a text database. An item (here, a word) *x*_*i*_ has the probability *P*(*x*_*i*_|*x*_*i*−(*n* − 1)_, …, *x*_*i*−1_). The text database was extracted from the Leipzig Corpora Collection, a ready to use corpora [33]. It contains a word frequency list as well as a word bi-grams list (co-occurrences as next neighbors) containing observed frequency counts, which were generated from approximately 1 million sentences publicly accessible.

Here, an *n*-gram of size 2 (also called bi-gram) was utilized, i.e., next word candidates were weighted according to the probability on the word level.

**Fig 2:**
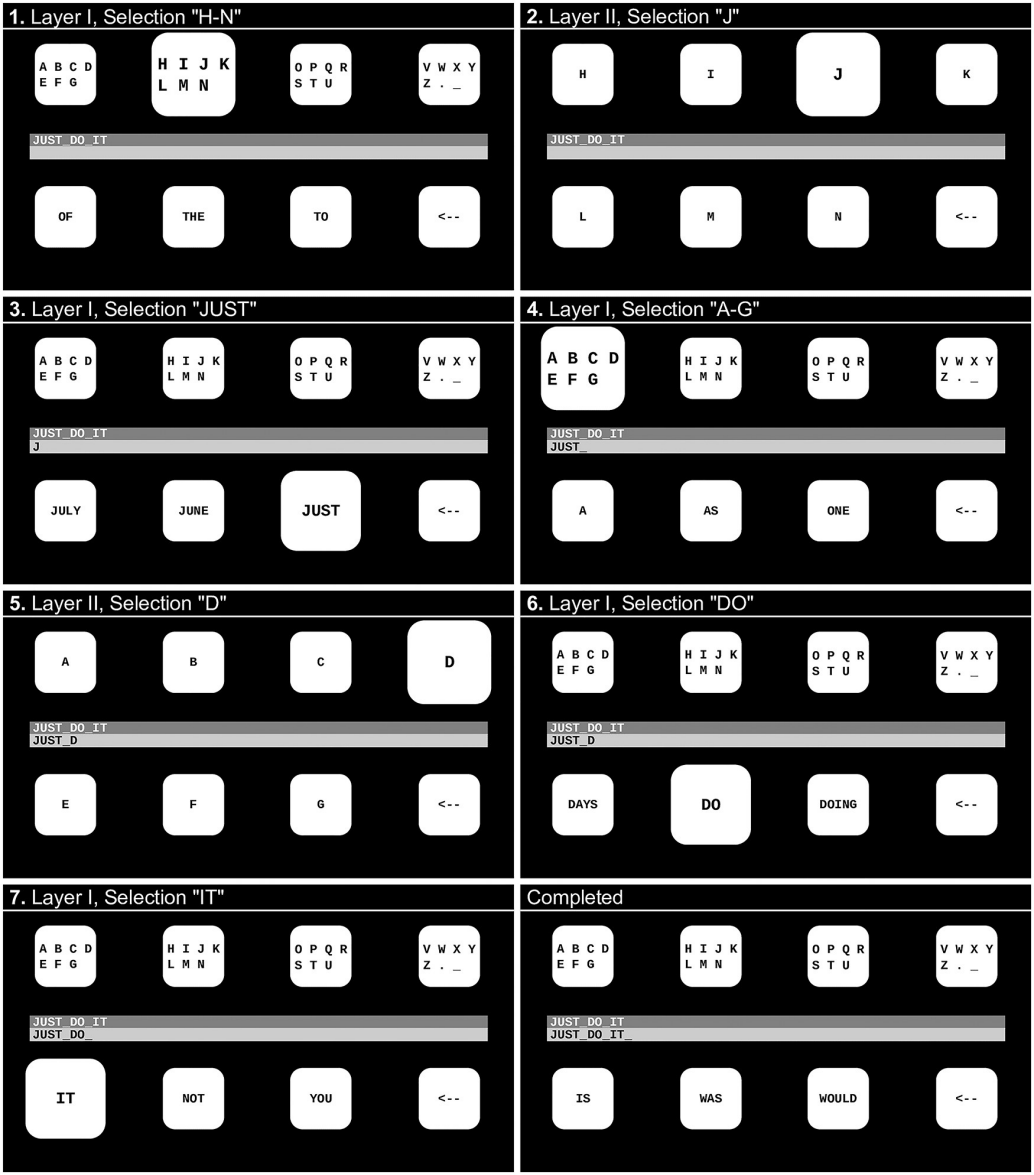
Writing a sentence with the dictionary-driven speller. A participant is writing JUST_DO_IT in seven steps. Selection of individual letters required two steps: Initially the group containing the character was selected (Layer I), and then the desired character was selected (Layer II).

Structured query language (SQL), a query language for relational databases, was used to retrieve word suggestions from the Leipzig text database. Based on the already typed string, three word suggestions where extracted using SQL statements. First, all co-occurrence pairs, including the previously typed word and the words beginning with the already typed part of the current word, were ordered according to their frequency. If this yielded less than three candidates, the suggestions were complemented with the word frequency list (independent of the precedent word), i.e., the most frequent words matching the already typed string were added. The speller presented eight selection options, arranged in a 2 × 4 matrix format (Fig 2). One of two layers was shown. In Layer I, the first row contained 28 characters (26 letters, underscore and full stop character) divided into four groups of seven characters. The second row contained the three dictionary suggestions as well as a correction option (undo the previous command). By selecting one of the group boxes from the first row, Layer II was presented, which allowed for the selection of individual characters.

The copy-spelling sentence and the user output were presented in the center of the screen. If the classifier produced an output command, audio and visual feedback were provided: The size of the selected box increased for a short time and a sound file, voicing the selected command, was played.

The functioning of the dictionary-driven spelling application is illustrated in Fig 2.

## Results

### Off-line performance

**Fig 3:**
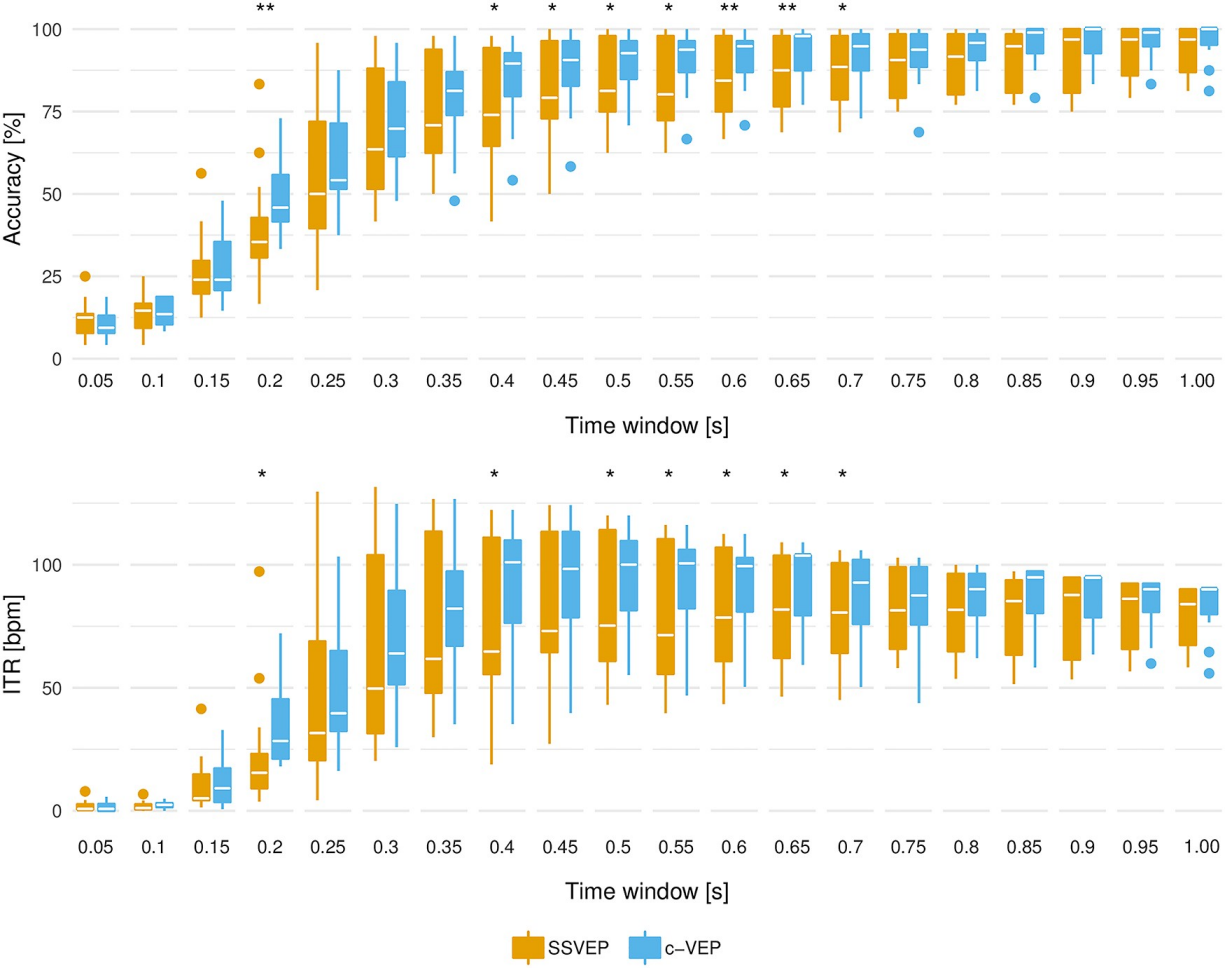
Comparison of SSVEP and c-VEP methods. ITRs and accuracies of all participants are presented. The asterisks mark statistical significance determined with paired sample t-tests (*p < 0.05 and **p < 0.01).

Optimal time windows and ITRs were calculated using off-line leave-one-out cross-validation. As expected, the highest ITRs were achieved with different time windows for each user. The time window yielding maximal ITR, which was used as minimal time window in the on-line experiment, as well as the corresponding maximum ITR are listed in Table 1. Fig 3 shows off-line ITRs and accuracies across all participants for a time window length up to 1.05 s with an interval of 50 ms. On average, participants reached a theoretical maximal ITR of 100.90 bpm and 95.21 bpm with the optimal time window for the c-VEP and SSVEP paradigm. However, the difference between the paradigms was not statistically significant (t = 1.488, p = 0.165).

**Table 1:** Information transfer rate (ITR) in bits/min for each participant for the c-VEP and SSVEP system.

	c-VEP				SSVEP			
Subject	Cl. Time	Training	Words	Sentence	Cl. Time	Training	Words	Sentence
S01	0.50 s	97.99	100.6	83.9	1.35 s	76.60	32.8	42.2
S02	0.35 s	126.71	76.5	36.2	0.30 s	131.58	70.7	62.4
S03	0.40 s	122.18	107.8	62.1	0.45 s	124.14	98.8	78.6
S04	0.60 s	82.40	85.9	58.6	0.90 s	94.74	45.5	53.0
S05	0.50 s	108.07	101.7	49.1	0.45 s	117.97	92.5	51.2
S06	0.55 s	104.58	107.6	59.9	0.80 s	95.03	91.2	41.1
S07	1.00 s	76.58	69.8	43.8	0.45 s	71.10	73.2	47.7
S08	0.45 s	111.80	111.0	89.6	0.65 s	79.93	76.7	77.3
S09	0.90 s	63.54	66.3	44.0	0.95 s	66.20	32.7	30.4
S10	0.80 s	85.09	84.2	46.1	0.45 s	72.45	88.4	63.4
S11	0.35 s	120.08	111.9	64.8	0.25 s	117.58	135.3	96.4
S12	0.45 s	111.80	88.5	47.3	0.45 s	95.21	62.9	43.4
Mean	0.57 s	109.00	92.65	57.11	0.62 s	95.20	75.1	57.3

Provided are the optimal time window determined in the training (leave-one-out cross-validation), the corresponding training ITR and the ITR achieved in the spelling tasks. The words column lists the mean ITR of the single word spelling tasks BRAIN and BCI.

### On-line spelling performance

The on-line performance was evaluated utilizing the output command accuracy, the ITR, as well as the output characters per minute (OCM) which measures typing speed by dividing the total number of output characters by the time needed to type them [14]. Measuring the OCM therefore takes into account that all errors are corrected. Tables 1, 2 and 3 summarize spelling results across all participants for each paradigm and spelling task.

**Table 2:** c-VEP spelling task accuracies and output commands per minute (OCM).

Subject	Sentence	Accuracy	OCM	Word	Accuracy	OCM
S01	THE TRAIN WAS OVERCROWDED	97	20.8	BCI, BRAIN	100	16.8
S02	THAT WAS AN INTERESTING LECTURE	100	12.1	BCI, BRAIN	100	12.7
S03	THE BOOK IS WAY TOO BORING	100	17.9	BCI, BRAIN	100	18.0
S04	I DO NOT LIKE TO EAT FISH	96	24.1	BCI, BRAIN	100	14.3
S05	DID YOU EVER DRIVE A SKATEBOARD	92	16.4	BCI, BRAIN	96	16.8
S06	THE RECORDING IS REALLY BAD	94	19.0	BCI, BRAIN	100	17.9
S07	I DO NOT LIKE THIS MUSIC AT ALL	92	15.9	BCI, BRAIN	100	11.6
S08	DOGS ARE NOT ALLOWED	100	37.3	BCI, BRAIN	100	18.5
S09	I WILL GO SWIMMING TOMORROW	84	16.7	BCI, BRAIN	94	10.9
S10	I NEED TO BUY A NEW TOOTHBRUSH	88	13.2	BCI, BRAIN	96	13.9
S11	THE SHOP WAS CLOSED ALREADY	100	20.8	BCI, BRAIN	100	18.7
S12	THE DOG BARKED LOUDLY	85	12.3	BCI, BRAIN	96	15.1
Mean	Mean length: 26.7 characters	94.0	18.9	4 characters	98.5	15.4

Provided are the specific spelling task, corresponding command accuracy, and output commands per minute (OCM) for each participant.

**Table 3:** SSVEP spelling task accuracies and output commands per minute (OCM).

Subject	Sentence	Accuracy	OCM	Word	Accuracy	OCM
S01	HE IS AFRAID OF HORSES	100	11.9	BCI, BRAIN	100	5.5
S02	I WANT TO BECOME A BUSDRIVER	100	16.2	BCI, BRAIN	87	12.3
S03	I WOULD LIKE TO PLAY THE CELLO	100	32.8	BCI, BRAIN	100	16.5
S04	I DO NOT SPEAK FINNISH	100	18.5	BCI, BRAIN	100	7.6
S05	ALL OF THE PHOTOS WERE BLURRED	95	14.2	BCI, BRAIN	100	15.4
S06	I COULD EAT PIZZA EVERYDAY	93	10.2	BCI, BRAIN	100	15.2
S07	I USUALLY FALL ASLEEP IN THE CINEMA	95	17.1	BCI, BRAIN	100	12.2
S08	THAT WAS A NICE MOVIE	100	28.5	BCI, BRAIN	96	12.6
S09	THEY OWN A BLACK CAT	91	8.0	BCI, BRAIN	96	5.6
S10	MY BIKE HAS NOT BEEN STOLEN	93	26.0	BCI, BRAIN	93	15.7
S11	I WANT TO LISTEN TO THE RADIO NOW	95	22.5	BCI, BRAIN	100	22.5
S12	DID YOU GO TO SCHOOL TODAY	94	14.1	BCI, BRAIN	100	10.5
Mean	Mean length: 26.8 characters	96.3	18.3	4 characters	97.6	12.6

Provided are the specific spelling task, corresponding command accuracy, and output commands per minute (OCM) for each participant.

For the single word spelling tasks (BCI and BRAIN), the average ITR was 92.65 and 75.06 bpm for the c-VEP and SSVEP system; the difference between the paradigms was significant (t = 2.503, p = 0.029). As expected, these values were lower than the off-line values because of the threshold criterion described in the previous section.

For the sentence spelling tasks, no statistically significant difference between the paradigms was found; ITRs of 57.11 and 57.26 bpm were achieved for the c-VEP and SSVEP paradigm.

Due to the *n*-gram prediction model, the average OCM was higher in the sentence spelling tasks in comparison to the word spelling tasks for the two tested paradigms. For the single word spelling tasks, 15.43 and 12.63 OCM were reached; for the sentence spelling tasks, 18.89 and 18.31 OCM were achieved with the c-VEP and SSVEP paradigm.

### Questionnaire results

**Fig 4:**
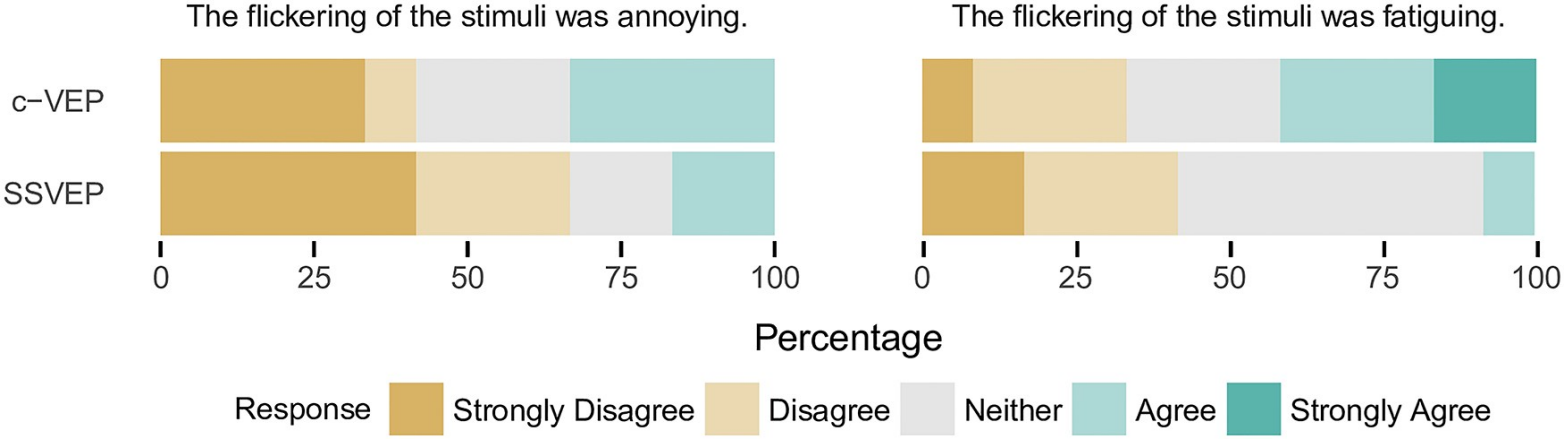
Results from the user questionnaires. Responses were given on a 1-5 Likert scale, 1 indicating strong disagreement and 5 indicating strong agreement.

Further, the results from the questionnaires are depicted in Fig 4. For an overview of the pre-and post-questionnaire answers, see also Table 4. The subjective impressions regarding fatigue level and annoyance were measured using a five-point Likert scale [34], where “1” indicated the strongest degree of disagreement and “5” the strongest degree of agreement.

**Table 4:** Questionnaire answers.

Subject	Gender	Age	SSVEP annoying	c-VEP annoying	SSVEP fatiguing	c-VEP fatiguing
1	f	25	3	2	2	4
2	m	24	1	4	1	3
3	m	30	2	3	1	1
4	m	23	3	3	3	3
5	f	23	2	2	2	3
6	f	21	2	1	1	1
7	m	22	3	4	3	2
8	m	25	1	2	1	1
9	f	25	4	5	4	4
10	f	24	3	3	4	4
11	f	22	3	4	1	1
12	f	21	3	5	2	4
Mean		23.75	2.50	3.17	2.08	2.58
Range		21-25	1-4	1-5	1-4	1-4

Provided are the answers collected from the pre- and post-questionnairies. In questions that were answered on a 1-5 Likert scale, 1 indicates strong disagreement and 5 indicates strong agreement.

The majority of the users did not find the flickering annoying or fatiguing. Overall, answers regarding the user-friendliness were slightly more positive for the SSVEP paradigm. Five out of twelve participants stated that they found the flickering of the c-VEP system annoying. In contrast, only one participant found the SSVEP to be annoying. In respect to the subjective level of fatigue, the SSVEP paradigm yielded better results. Four participants found the c-VEP flickering fatiguing, but only two participants stated that the SSVEP flickering caused fatigue.

A spearman rank correlation test was performed to investigate if the tiredness affected the spelling performance. Neither for the SSVEP experiment nor for the c-VEP experiment a significant relationship was found between the subjective level of tiredness and the mean ITR of the sentence spelling task (*r*_*s*_ = −0.43, n.s for SSVEP and *r*_*s*_ = −0.44, n.s. for c-VEP).

## Discussion

The main purpose of the presented study was to investigate methods of dynamic gaze classification time windows for time synchronous VEP BCIs. The proposed methods lead to a more natural user-BCI interaction. To demonstrate the robustness of the approach, a dictionary-driven spelling application was tested with the SSVEP and the c-VEP paradigm. In this sense, the study also provides a direct comparison between c-VEP and SSVEP stimulation, both in terms of performance and user-friendliness.

The comparison of the two stimulation approaches indicates that c-VEP slightly outperforms SSVEP in terms of ITR, while SSVEP is preferred by most users in terms of user-friendliness, see Figs 3 and 4.

For the SSVEP paradigm, the flickering was realized by sinusoidally modulating the transparencies. This allowed a slightly more subtle visual stimulation in comparison to the c-VEP flickering which switched from full illumination to no illumination in correspondence to the code patterns. Indeed, a slight difference regarding the subjective level of annoyance and fatigue caused by the flickering is evident from the questionnaires (Fig 4). Regarding the user-friendliness, most participants seemed to favor the SSVEP paradigm. It should be noted that a more subtle stimulation could be achieved by utilizing higher carrier frequencies for the c-VEP paradigm as well [35]. On the other hand, faster rate flickering could lead to a lower performance for some users [36]. A similar effect has been observed for SSVEP stimuli as well [37]. For SSVEP BCIs, a more subtle stimulation could also be achieved by utilizing motion-based stimulation [38]. Further, Chien et al. [39] achieved promising results with little flickering sensations by employing a composition of red/green/blue 32 Hz/40 Hz flashing lights.

As can be seen in Fig 3, the variability between subjects (inter-subject variability) seems to be slightly higher for the SSVEP paradigm than for the c-VEP paradigm. A reason for this could be that the tested frequencies interfere with the natural brain activity. As stated by Bin et al. [12], narrow-band signals in the natural EEG (e.g. alpha and beta rhythms) are likely to interfere with low frequency SSVEP stimuli.

Here, a training session was utilized in both methods for template recording and parameter optimization. It should be noted that in general, c-VEP BCIs require a training stage to obtain templates. SSVEP BCIs on the other hand can be realized without training if desired, by utilizing sine- and cosine templates. Typically, for the recording of the c-VEP templates, only one code sequence is utilized for the training session. By shifting the recorded data templates, the required amount of classes can be generated. In this study, however, templates were recorded and averaged separately for each c-VEP class. This was done for the following reasons. It facilitates an ensemble-based classification approach. Further, as demonstrated by Nagel et. al, monitor latencies differ depending on the position of the target [40]; using all targets to create one single template would require a correction of these latencies.

One of the key parameters for BCI performance is the time window used for the classification of the signals. User variability justifies user-dependent selection of a minimum value for the minimum classification time interval [31]. For the c-VEP paradigm, typically, this interval is determined by the cycle length; e.g., for 63 bit m-sequence stimuli with 60 Hz monitor refresh rate, a time window of 1.05 seconds can be utilized [10, 41]. SSVEP BCIs have been used with time windows as low as 0.3 s [5]. In some studies larger classification windows were incorporated to improve robustness of the system. For example, to outbalance the lower signal-to-noise-ratio with dry electrodes, Spüler [41] utilized larger classification windows in a c-VEP BCI by averaging over multiple trials. Similarly, in previous studies, we incorporated large classification windows to deal with age-related inter-subject variability in users [8, 42].

For the system presented in this paper, a dynamic threshold-based time window approach was utilized. Two conditions needed to be met before an output was produced:

1. The distance class yielding maximum correlation needed to surpass the other correlations by a certain threshold amount,
2. the time window for classification needed to be sufficiently long.

Here, the minimal time window was set user-specifically based on the ITR using the recorded training data. A similar approach was utilized in our previous study, where the time window was determined in relation to off-line accuracy [31].

Interestingly, although, good correlation properties of the m-sequence require a full stimulation cycle (here 1.05 s), high accuracies were achieved with an incomplete code sequence (see Fig 3). On average, the optimal classification time window in terms of off-line ITR was 0.57 s (ranging from 0.35 s to 1 s) for the c-VEP paradigm and 0.62 s (ranging from 0.25 s to 1.35 s) for the SSVEP paradigm. Hence, despite the comparably low number of targets, high ITRs were achieved. This can also be attributed to the utilization of ensemble methods, which can significantly increase system speed [5]. Additionally, for the SSVEP paradigm a filter bank approach as proposed by Chen et al. [17] was utilized to enhance target discrimination. Similar methods could also enhance the classification accuracy for the c-VEP approach. Therefore, our future work will focus on investigating suitable cut-off frequencies for the c-VEP paradigm.

Regarding the copy spelling phase, the number of output characters per minute was on average significantly higher for the sentence spelling tasks, in comparison to the word spelling tasks where no dictionary suggestions were used (see Tables 2 and 3). This demonstrates the robustness of the proposed time window approach as well as the effectiveness of the implemented word suggestion methods. It should be noted, that participants did not always use the dictionary whenever they had the chance to do so (suggestions were simply overseen). For this reason, in some cases, the single word OCM was higher in comparison to the sentence task. Improvements could be made in regards to the arrangement of the GUI targets to make the suggestions more prominent. For some participants, a longer gaze shifting phase when suggestions are presented could also be helpful. It should further be noted, that participants used the system for the first time. More experience with the GUI could improve OCM as well.

Overall, there was surprisingly little performance difference between the two stimulation modalities. The c-VEP stimulation patterns yielded slightly higher off-line ITRs and significantly higher ITRs in word copy spelling tasks. In on-line sentence spelling, the speed difference becomes negligible. This is due to the fact that in sentence spelling, usually larger search phases are required to find the next letter or word to select.

## Conclusion

A dynamic classification time window approach for time synchronous VEP BCIs was proposed. The optimal time window was determined individually deduced from a training session which was also used for the generation of templates and spatial filters. An 8-target spelling application utilizing *n*-gram-based word suggestions was used to evaluate the usability of the developed methods. Twelve participants tested the system in on-line spelling tasks with the SSVEP and the c-VEP paradigm. The presented study demonstrates the robustness of the proposed approach. All participants completed sentence spelling and word spelling tasks with accuracies well above 90% for the two paradigms. Ensemble-based classification strategies were employed in both cases. The proposed methods were equally effective for c-VEP and SSVEP based systems in terms of ITR; mean ITRs of approximately 57 bpm were achieved in both cases. Nevertheless, in word spelling tasks, the c-VEP system (mean word spelling ITR 92.7 bpm) outperformed the SSVEP system (ITR 75.1 bpm). In terms of user-friendliness however, the SSVEP paradigm was preferred by most participants. The results suggest that the stimulation pattern (SSVEP vs. c-VEP) could be selected based on the user preference. In terms of speed, the optimal paradigm could be determined individually for each user in a short training session. However, the perceived level of user-friendliness should also be taken into account, as it might be more relevant for end users than pure system speed.
